# Recruiting for local needs

**Published:** 2018-07-31

**Authors:** Shantanu Das Gupta

**Affiliations:** 1 Deputy General Manager: Marketing & Projects, Dr Shroff's Charity Eye Hospital, India.

Dr Shroff's Charity Eye Hospital is a network of eye hospitals consisting of a main tertiary hospital in New Delhi, India and five smaller hospitals in surrounding areas. In 2012, we faced a significant shortage of trained allied health personnel (support workers), which prevented us from moving to a high-volume eye surgery model.

Because the candidates available locally were few and very inadequately trained, we decided to create our own workforce that would have the necessary skills and would be aligned to the mission, vision and values of our institution. In 2014, with the support of Lavelle Foundation for the Blind, we launched our own certified ophthalmic paramedic programme.

## Deciding who to train

Rural Indian society in North India is deeply patriarchal, with girls considered to be less valuable than boys, and early marriage is a common cultural practice. To address this, the programme was limited to women from underprivileged backgrounds aged 18–21 who had completed high school and lived near one of the hospitals. We felt that the programme had the ability to not only improve the economic status of the family but would empower the women in the long run. Candidates are selected after a written test, a personal interview and a meeting with their parents. A five-month foundation course at the tertiary hospital is then followed by 19 months of on-the-job training at the hospital nearest to them. It all culminates in an internal certification exam and a formal graduation. Different modules train women as vision technicians, nursing assistants, operating theatre assistants, medical record administrators, front office personnel and patient counsellors. Other modules currently in development include optical services, housekeeping, stores & purchasing and basic accounting.

At the start of the programme, the big questions were:

- Would we get support from the community and the families?
- What proportion would leave after we had invested in their training and employment?
- Would this approach really help us to reach out to more patients and reduce avoidable blindness?

As expected, the recruitment of candidates was difficult initially, as it challenged cultural expectations. However, the reputation of the hospital and the ambition of the young women meant that 15 women were enrolled in the first year. After graduation, they became the best ambassadors for the course and the career pathway, which is evident in the fact that we currently have three intakes per year of 30 students each.

As a result of the programme, we have seen a 64% increase in the number of outpatients (from 250,000 to 400,000) and a 62% increase in the number of operations (from 18,000 to 29,000).

**Table:** 

	2013–2014	2016–2017	% growth
**Outpatients**	245,357	402,429	64%
**Operations**	17,584	28,543	62%

Out of the 305 women trained to date, 30 (10%) have left Shroff at various stages, mainly because of moving away from the area after marriage.

The initiative is guided by a 5-year plan that links recruitment and training to our strategic goals on volumes. As a result, we have been able to assure jobs for all our graduates over the next five years, provided that the current growth in patient volume is maintained.

The labour market pressures we faced in 2012 forced us to take radical action. Before, we were just a hospital providing quality eye services. Today, we offer valuable employment that empowers young women and brings local communities closer to us.

